# Oral Mucosal Ulceration Caused by the Topical Application of a Concentrated Propolis Extract

**DOI:** 10.1155/2014/307646

**Published:** 2014-09-09

**Authors:** Yuniardini Septorini Wimardhani, Anandina Irmagita Soegyanto

**Affiliations:** Department of Oral Medicine, Faculty of Dentistry, Universitas Indonesia, Jakarta 10430, Indonesia

## Abstract

Propolis is a resinous mixture that is collected by honey bees from tree buds, sap flow, and other botanical sources. Propolis has been extensively used in medicine, dentistry, and cosmetics; however, unwanted effects have been reported. This paper reports a case of oral mucosal burn in a 50-year-old patient, who used an overnight application of concentrated propolis to overcome a throbbing pain in the right upper posterior mucosa. The patient was otherwise healthy and was not receiving any medication. She presented with painful shallow multiple irregular ulcers measuring 0.3–1 cm in diameter that were located on the right buccal mucosa and hard palate mucosa, in addition to the gingival mucosa surrounding tooth 17. Propolis-induced oral mucosal burn was diagnosed. The ulcer cleared after the prescription of tetracycline mouthwash, accompanied with Doloneurobion. The patient was further treated with carbamazepine to address the persistent throbbing pain in the affected area, which was suspected to be trigeminal neuralgia. This report provides another alert to clinicians about the potential adverse effects of propolis use for the treatment of oral diseases, despite its natural origin.

## 1. Introduction

Propolis is a Greek word that literally means “in front of the city,” and it is sometimes referred to as bee glue. It is collected by honey bees to construct their hives and serves as waterproof and protection material against invaders [1]. The chemical analysis of propolis has revealed at least 300 compounds as its constituents [2]. It is a complex mixture containing resinous and balsamic compounds (55%) as its major constituents. The remaining constituents are beeswax (30%), essential oils (10%), bee pollen (5%), and organic compounds (5%; phenolic, esters, and flavonoids) [3]. These components are collected from tree buds, sap flow, and other botanical sources. The location of plants, climate, and environmental conditions have an important role in determining the ratio and concentration of the components of propolis [1, 4].

For many years, propolis has been considered as a traditional herbal medicine that heals various diseases [5]. Propolis has been extensively used in medicine, dentistry, and cosmetics.* In vitro* and* in vivo* animal studies of propolis have inferred a number of its biological activities. For example, it exhibits astringent, antiseptic, anesthetic, anti-inflammatory, antibiotic, antifungal, antiviral, antioxidant, immunomodulator, and antineoplastic activities [5–8]. However, clinical studies of propolis for oral diseases in humans remain limited [9–11]. Despite the benefits of using propolis in medicine and dentistry, allergic reactions due to propolis have also been reported [12, 13]. A recent study described 22 cases of oral lesions induced by the use of propolis, indicating that the improper use of propolis may have serious adverse effects on the oral mucosa [14].

Although several published reports have described adverse reactions to propolis [12, 13], we recently documented a new case related to its use. Here, we report a case of a woman who developed oral mucosal ulcers after the topical use of concentrated propolis on a painful dental area.

## 2. Case Presentation

**Figure 1:**
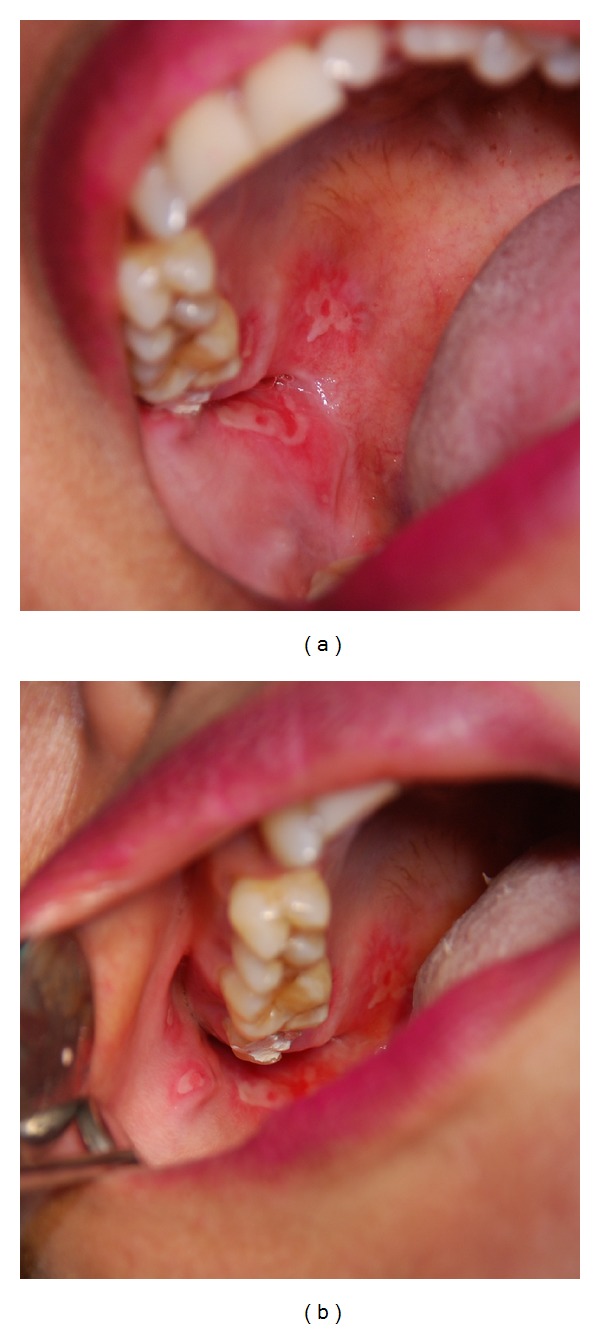
Shallow multiple irregular ulcers ranging from 0.3 to 1.5 cm in diameter with erythematous border, located on the right buccal mucosa, palatal mucosa, and gingival mucosa surrounding tooth 17.

**Figure 2:**
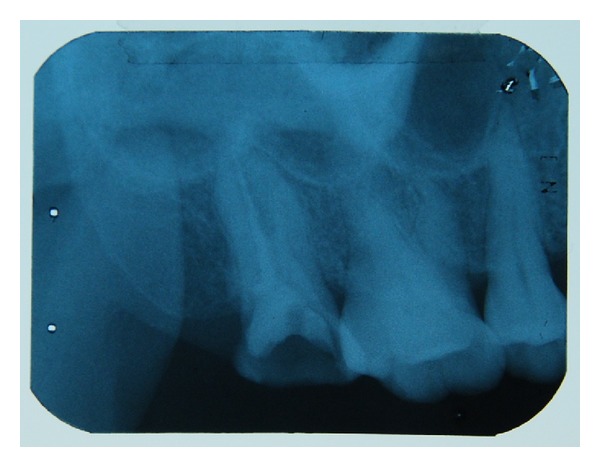
Dental radiograph showing the wide and deep caries lesion on tooth 17, with unfinished endodontic treatment. No periapical lesion was observed.

A 50-year-old female patient was referred to the Oral Medicine Clinic, Faculty of Dentistry, Universitas Indonesia, complaining of a painful lesion located on her upper right buccal mucosa, in addition to the palatal mucosa. She had experienced throbbing pain in the mucosal tissue around tooth 17 a few days earlier. She reported the self-application of a cotton roll that had been damped in propolis to relieve the pain in the mucosal area. The cotton roll was left in contact with the mucosa overnight. She noticed the eruption of a painful oral lesion the next morning, which caused difficulty in eating. The eruption of the lesion was not accompanied by any systemic symptoms, and no other body areas were involved. She also reported the daily consumption of propolis diluted in her drinking water. A review of her medical history revealed an allergic history to chloramphenicol and occasional gastric pain. Otherwise, the patient was healthy and was not under any medication. A clinical examination revealed multiple shallow and irregular ulcerations on the right buccal mucosa, the right hard palate mucosa, and the gingival area surrounding tooth 17. The size of the ulcers ranged from 0.3 to 1.5 cm in diameter (Figure 1). Tooth 17 was in the middle of endodontic treatment for wide and deep caries lesion, and the radiograph showed no periapical lesion (Figure 2). Palpation and percussion of tooth 17 were within normal limits. However, the patient considered having tooth 17 extracted. Furthermore, enlarged and painful submandibular lymph nodes were noted on palpation and were possibly the result of inflammation related to tooth 17. A working diagnosis of propolis-induced mucosal burns was made. The patient was advised to discontinue propolis use and was prescribed tetracycline mouthwash three times daily for 3 days and Doloneurobion twice daily for 7 days to manage the pain. She was advised to make a followup consultation after 5 days.

On the followup consultation, extra oral examination indicated a normal appearance of the affected area. Tooth 17 had been extracted by a different department, because the patient believed that it was the cause of the throbbing pain. Clinically, there was a healing extraction socket of tooth 17 and healing of the ulcerated area that was seen as the erythematous area. Pain related to the postulcerated area had mainly resolved; however, she reported persistent throbbing pain in the area where tooth 17 had been extracted. No submandibular lymphadenopathy was observed during this visit. Our department suspected trigeminal neuralgia as the cause of the throbbing pain. The patient was prescribed a gauze mucosal compress with 0.05% chlorhexidine gluconate three times daily for 3 days to heal the oral mucosa, in addition to 100 mg carbamazepine twice daily for 5 days. The patient was asked to return for a followup consultation in 5 days.

**Figure 3:**
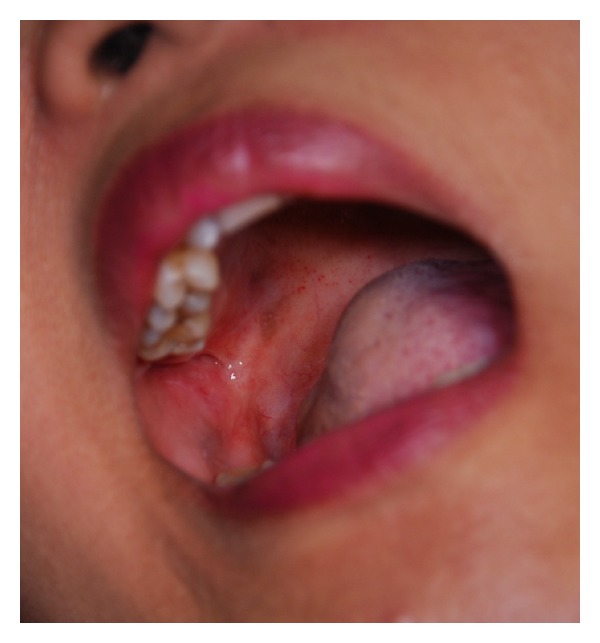
The previously ulcerated oral mucosa healed after 5 days of treatment with tetracycline mouthwash.

On the final consultation, the erythematous area was completely healed, and the rest of the mucosa appeared normal, with the socket of tooth 17 healing after extraction (Figure 3). The patient reported no pain related in the postulcerated area. However, the throbbing pain in the area of tooth 17 was noted as a “funny feeling.” A 100 mg dose of carbamazepine was prescribed twice daily for 2 weeks. The ulcer was declared to be healed at this visit, and the patient was scheduled for a followup consultation 2 weeks later for the further evaluation of the suspected trigeminal neuralgia.

## 3. Discussion

The various biological benefits of propolis have resulted in it being widely used in medicine, including dentistry [10, 11]. Many* in vitro* and* in vivo* studies on propolis have been completed, with several clinical trials on humans showing its beneficial use as an active ingredient for the treatment of eosinophilic ulcers, as an antimicrobial for gingivitis patients, as a component of pulp capping materials, and as an antifungal for patients with denture stomatitis [15–17]. However, adverse reactions to propolis have also been reported and described in the published literature [12, 13]. A 50-year-old female patient with an oral lesion due to the topical application of propolis was described in this case report.

After careful analysis of the nature of the lesion, our patient was diagnosed as having oral mucosal burns following direct contact with concentrated propolis. The patient in this case report decided to compress the mucosa with a cotton roll damped with concentrated propolis to ease a throbbing pain in the affected area overnight. The eruption of the lesion in the contact area happened approximately 8 hours after the application of propolis, without any systemic conditions. The high concentration of ethanol component (50–70%) in the propolis extract might be the cause of the mucosal burn. The propolis extract had been subject to a series of extraction processes that use highly concentrated alcohols before it is made publically available [1, 13]. This high alcohol component might have caused the damage to the oral mucosa in this patient [18]. In addition, keeping a cotton roll in the mouth for several hours might have also caused trauma to the oral mucosa. The ulcer completely healed after 12 days, following the cessation of propolis use and the prescription of appropriate antibiotics with anticollagenolytic effects, in parallel with antiseptics and supportive measures.

Although cases of allergic reactions to the topical application of propolis have been reported, we did not suspect that this was the case for our patient [19]. Our patient had a long history of propolis use in her daily life and had been adding propolis to her drinking water, with no adverse effects. Many reports state that the median time for lesions related to allergy to propolis occurs after 2.5 days (range: 0–15 days). In contrast, our patient developed the ulcer approximately 8 hours after propolis application [19–22]. Studies on the allergic potential of propolis have revealed that it should not be used as a topical product due to its high sensitizing characteristics [22]. The minor constituents in propolis, such as 3-methyl-2-butenyl caffeate and phenyl-ethyl caffeate, are major allergens, in addition to benzyl-salicylate and benzyl-cinnamate [23]. We did not order a patch test to check for a possible allergic reaction of our patient to propolis. Therefore, we could not confirm whether the oral ulceration was due to a propolis allergy within 8 hours of exposure [19]. However, possible allergic contact mucositis that was facilitated by injury to oral mucosa could be postulated.

This case report provides another alert to clinicians about the potential adverse effect of propolis when used to treat oral diseases, as some propolis applications may have serious negative effects. Although there is an increasing global trend in the use of propolis for medication, the important discovery of its beneficial roles should be in parallel with research undertaken to specifically define its application in many areas of dentistry. Careful consideration should be given before using propolis to treat oral diseases, as many clinical complications might arise, despite its natural origin [24].
